# The association between BMI and 90-day mortality in patients with and without diabetes seeking care at the emergency department

**DOI:** 10.48101/ujms.v126.7590

**Published:** 2021-09-16

**Authors:** Per Wändell, Axel C. Carlsson, Anders Larsson, Olle Melander, Torgny Wessman, Johan Ärnlöv, Toralph Ruge

**Affiliations:** a Department of Neurobiology, Care Sciences and Society, Karolinska Institutet, Huddinge, Sweden; b Academic Primary Health Care Centre, Stockholm Region, Stockholm, Sweden; c Department of Medical Sciences, Uppsala University, Uppsala, Sweden; d Department of Emergency and Internal Medicine, Skånes University Hospital, Malmö, Sweden; e Department of Clinical Sciences Malmö, Lund University & Department of Internal Medicine, Skåne University Hospital, Malmö, Sweden; f School of Health and Social Studies, Dalarna University, Falun, Sweden

**Keywords:** Diabetes, BMI, mortality, triage level, emergency department

## Abstract

**Background:**

The impact of body mass index (BMI) on mortality varies with age and disease states. The aim of this research study was to analyse the associations between BMI categories and short- and long-term mortality in patients with or without diabetes seeking care at the emergency department (ED) with acute dyspnoea.

**Population and methods:**

Patients aged ≥18 years at ED during daytime on weekdays from March 2013 to July 2018 were included. Participants were triaged according to the Medical Emergency Triage and Treatment System-Adult score (METTS-A), and blood samples were collected. Totally, 1,710 patients were enrolled, with missing values in 113, leaving 1,597 patients, 291 with diabetes and 1,306 without diabetes. The association between BMI and short-term (90-day) and long-term (mean follow-up time 2.1 years) mortality was estimated by Cox regression with normal BMI (18.5–24.9) as referent category, with adjustment for age, sex, METTS-A scoring, glomerular filtration rate, smoking habits and cardiovascular comorbidity in a fully adjusted model. The Bonferroni correction was also used.

**Results:**

Regarding long-term mortality, patients with diabetes and BMI category ≥30 kg/m^2^ had a fully adjusted Hazard Ratio (HR) of 0.40 (95% confidence interval [CI]: 0.23–0.69), significant after the Bonferroni correction. Amongst patients without diabetes, those with underweight had an increased risk but only of borderline significance, whilst risks in those with overweight or obesity did not differ from reference.

Regarding short-term mortality, risks did not differ from reference amongst patients with or without diabetes.

**Conclusions:**

We found divergent long-term mortality risks in patients with and without diabetes, with lower risk in obese patients (BMI ≥ 30 kg/m^2^) with diabetes, but no increased risk for patients without diabetes and overweight (BMI: 25–29.9 kg/m^2^) and obesity.

## Introduction

Globally, body mass index (BMI) has increased during the last few decades (1), with an increasing rate of overweight and obesity, although a plateau phase seems to be reached in many high-income countries (2). Obesity has been proposed as the main upstream driver of cardiometabolic risk factors and associated outcomes (3), such as hypertension, diabetes and myocardial infarction (MI) (4). Moreover, obesity is regarded as one of the leading risk factors for mortality in the world (5). Despite that overweight and obesity are associated with an increased risk of mortality (6), cardiovascular mortality, especially coronary heart disease (CHD), has decreased in Western countries (7), including in Sweden (8), during the last few decades.

The association between BMI and mortality has been explored in many articles. A J- or U-shaped association between BMI and both all-cause and cardiovascular mortality has been reported (9, 10). In a study of the risk of being among the lowest 5% in different anthropometrical measures, both BMI and percent body fat showed an increased mortality risk, whilst all measures showed an excess mortality risk in the highest quartile (11).

A paradoxical risk association has been observed between BMI and mortality, particularly for patients with diabetes, with a higher mortality risk in the BMI range 18.5–24.9 kg/m^2^ (12). In a recent review, the lowest mortality risk was found for men in the BMI range 31–35 kg/m^2^, and for women in the BMI range 28–31 kg/m^2^ (13). However, the BMI-mortality paradox in diabetes is still controversial, with some results in agreement (14), and others questioning it (15, 16), and the impact of obesity on health can be very different according to age, gender, patient characteristics and the presence of comorbidities, as well as the state of acute or non-acute illness (17). Thus, the role of BMI in patient outcome, whether in long term or short term, is still not clear.

The aim of this study was to determine the effect of BMI on short- and long-term mortality rates in elderly acute ill patients, with or without diabetes, admitted to the emergency department (ED). As a model, we chose a cohort of patients admitted to the ED because of acute dyspnoea, the ‘Acute Dyspnea Study’ (ADYS) (18), known to include severely ill patients with a high level of comorbidity.

## Methods

### Population and methods

Patients with acute dyspnoea admitted to the ED of SUS Malmö were included in the ‘Acute Dyspnea Study’ (ADYS) between March 2013 and January 2019. Patients ≥18 years who presented to the ED during daytime, 6:45 AM to 4:30 PM working days with acute dyspnoea as their main complaint, were asked for consent to take part in the study by a research nurse. Critically ill patients who directly were transferred from the resuscitation room to an intensive care unit (ICU) were excluded, as were patients with lower degrees of consciousness as these patients were not able to give consent nor able to partake in answering the questionnaire. Vital parameters were registered as were medical triage priority level according to the validated Medical Emergency Triage and Treatment System (METTS). A total of 1,710 patients were enrolled, in which 113 of these were excluded because of missing values, leaving 1,597 patients for further analyses. Body mass index data were present amongst 1,553 patients, and the 44 missing values were imputed for further analyses (see below). Of the included 1,597 patients, 291 patients had diabetes and 1,306 were without diabetes. Patients with diabetes in this study included both patients with type-1 and type-2 diabetes, and patients were only defined as diabetic if they had been previously diagnosed with diabetes in the electronic patient record.

After inclusion, all patients were interviewed by a research nurse about their health, medication, symptoms and social situation according to a standardised and approved questionnaire. Patients were questioned regarding smoking habits and categorised as non-smokers, former smokers (cessation 1 month ago or longer) or active smokers (regularly smoking the past month or longer), disease history associated with dyspnoea (i.e. congestive heart failure, chronic obstructive pulmonary disease (COPD), asthma, coronary artery disease, atrial fibrillation, restrictive lung disease, cancer, thromboembolic disease or rheumatic disease) and current medications. Information on weight and height was taken from the medical records if this was recently documented; otherwise, this information was collected at the ED by the research nurse. The research nurses reviewed the patient’s medical records in order to confirm the details with the support of senior physicians whenever uncertainties occurred. Originally METTS-A uses five clinical priority levels with increasing clinical priority: blue (lowest clinical priority – not life-threatening), green, yellow, orange and red (highest clinical priority – life-threatening). The lowest clinical priority level, blue, was not used in the clinical triage of the patients included here because of the local triage routines, with the lowest clinical triage priority here being green.

Body mass indexwas categorised into <18.5 kg/m^2^ (underweight), 18.5–24.9 kg/m^2^ (normal weight), 25–29.9 kg/m^2^ (overweight) or ≥30 kg/m^2^ (obesity) (19). High sensitivity C-reactive protein (CRP) was analysed using a particle-enhanced turbidimetric assay, and the creatinine level was analysed using an Integrated Database Management System (IDMS) calibrated enzymatic assay (20). Lactate and glucose were analysed with routine methods. These methods are accredited by Swedac and are included in the Swedish external quality assurance programme for CRP and creatinine, respectively, and are routinely used. Within an hour of presentation at ED, blood was sampled, serum and plasma were separated and subsequently stored at −80°C for future analysis. The assays were performed blinded without knowledge of clinical data. The Chronic Kidney Disease Epidemiology Collaboration (CKD-EPI) equation was used to estimate the glomerular filtrate rate (GFR).

### Ethical considerations

This study obtained ethical approval from ‘Regionala Etikprövningsnämnden EPN’, Lund, Sweden. Dnr 2014/82. All included patients provided their written informed consent to take part in the study.

### Statistics

Baseline values expressed as numbers, with percentages, or mean values with standard deviation (SD), and Chi-square tests or Analysis of variance (ANOVA) were used for comparisons. The association between the short-term mortality (90-day mortality) and long-term mortality (after total follow-up time of the study, 2.1 ± 1.5 years), respectively, and BMI categories was analysed by Cox proportional hazard model in univariate analyses and in models adjusted for age, sex (Model A), and the clinical triage scoring system (Model B), and cardiovascular co-morbidity (established coronary disease, heart failure and hypertension), smoking habits and GFR (Model C). We categorised the patients into those with or without diabetes, and performed interaction analyses. Individuals with normal BMI (18.5–24.9) was used as the referent category. We imputed values for BMI (missing in 44 patients), for the completeness of data for further analyses. We also performed a sensitivity analysis for patients with diabetes merging BMI levels 25–29.9 and ≥30 kg/m^2^ using Models A and C, as the Hazard Ratio (HR) showed similar values for both these categories.

The follow-up time stretched from the time of presentation at the ED to death within or the end of follow-up (90 days post-presentation and total mortality 2.1 ± 1.5 years). A *P*-value of <0.05 was considered to be statistically significant, and we also performed a Bonferroni correction (with 0.05 divided by 9 for statistical significance of outcomes using each group of models per patient group as a unit). The dataset was handled, and Cox proportional hazard models were all computed with IBM SPSS statistics 25.0 software (SPSS Inc., Chicago, IL).

## Results

### General data

Baseline data show that patients with diabetes were older, with higher mean BMI, and with higher lactate, glucose and creatinine levels (Table 1). Patients with diabetes also were more often admitted to hospital care, and more often were diagnosed with heart failure, CHD, stroke and hypertension than those without diabetes. Mortality, according to the BMI interval for 90-day mortality, shows the highest risk for diabetes patients in the normal BMI interval, that is, 18.5–24.9 kg/m^2^, with 21%, and for patients without diabetes in the underweight interval, that is, <18.5 kg/m^2^ with 22% (Table 2). There were no deaths reported amongst the two patients with diabetes in the BMI interval <18.5 kg/m^2^.

**Table 1:** Baseline characteristics of patients with acute dyspnoea, with and without diabetes seeking care at a hospital emergency department.

Variable	Diabetes (*n* = 291)	No diabetes (*n* = 1,306)
Female	44%***	58%
Age at survey (years)	74 (14)***	69 (19)
Body mass index (kg/m^2^)	30 (7)***	26 (6)
Systolic blood pressure (mmHg)	148 (27)	146 (29)
Diastolic blood pressure (mmHg)	80 (18)	82 (16)
Respiratory rate (frequency)	25 (7)	24 (7)
C-reactive protein (CRP, mg/L)	30 (53)	35 (64)
Lactate (mmol/L)	2.1 (1.2)***	1.7 (1.04)
Glucose level (mmol/L)	11.0 (5.9)***	6.9 (2.4)
Creatinine (µmol/L)	125.3 (100.6)***	93.0 (69.4)
*METTS-A triage*		
Red – most acute (%)	16	12
Orange (%)	32	31
Yellow (%)	49	50
Green – least acute (%)	4	7
Admitted to hospital care (%)	69***	46
Cancer (%)	24*	18
Chronic obstructive pulmonary disease (%)	29	30
Chronic heart failure (%)	53***	29
Coronary artery disease (%)	50***	24
Stroke (%)	17***	9
Hypertension (%)	63***	37
90-day mortality (%)	14	12

Missing data points were less than 4% for all included characteristics, except for diastolic blood pressure and lactate where around 8% of data points were missing. Means and standard deviations, or percentages

* *P* < 0.05

*** *P* < 0.001; for differences between individuals with or without diabetes.

**Table 2:** Data on a 90-day mortality by BMI categories in patients seeking care for dyspnoea at the emergency department with (*n* = 291) or without (*n* = 1,306) diabetes.

BMI categories	Patients with diabetes Number of mortality events or numbers at risk (%)	Patients without diabetes Number of mortality events or numbers at risk (%)
90-day mortality:		
BMI < 18.5 kg/m^2^	0/2 (0)	18/83 (22)
BMI 18.5–24.9 kg/m^2^	16/76 (21)	59/580 (10)
BMI 25–29.9 kg/m^2^	10/89 (11)	41/328 (13)
BMI ≥ 30 kg/m^2^	11/114 (10)	27/281(10)
Total mortality:		
BMI < 18.5 kg/m^2^	1/2 (50)	32/83 (39)
BMI 18.5–24.9 kg/m^2^	37/76 (49)	136/580 (23)
BMI 25–29.9 kg/m^2^	31/89 (35)	66/328 (20)
BMI ≥ 30 kg/m^2^	25/114 (22)	52/281(18)

BMI: body mass index.

### 90-day mortality

There was no significant interaction for the mortality between patients with and without diabetes (*P* < 0.07). Patients with diabetes and in the BMI intervals 25–29.9 and ≥30 kg/m^2^ showed no statistically decreased mortality risk (Table 3). Patients without diabetes had the highest risk in the BMI interval <18.5 kg/m^2^; however, it is not statistically significant after the Bonferroni correction (Bonferroni-adjusted *P*-level < 0.006).

**Table 3:** The associated risk between non-normal BMI and 90-day mortality in patients with or without diabetes seeking care at a hospital emergency department.

BMI categories	Patients with diabetes (HR, 95% CI)			Patients without diabetes (HR, 95% CI)		
	Model A	Model B	Model C	Model A	Model B	Model C
BMI < 18.5	-	-	-	2.11 (1.24–3.60)	2.11 (1.24–3.60)	2.33 (1.75–3.11)
BMI 18.5–24.9	Referent	Referent	Referent	Referent	Referent	Referent
BMI 25–29.9	0.54 (0.24–1.20)	0.68 (0.29–1.60)	0.54 (0.21–1.38)	1.23 (0.83–1.84)	1.23 (0.82–1.84)	1.22 (0.87–1.72)
BMI ≥ 30	0.46 (0.21–1.02)	0.50 (0.22–1.11)	0.50 (0.22–1.13)	1.20 (0.75–1.88)	1.23 (0.77–1.95)	1.24 (0.77–1.98)

BMI: body mass index; CI: confidence interval; Hazard Ratio (HR): .

Analyses include imputed data for missing values for BMI.

Model A includes age and sex; Model B includes Model A and METTS-A triage; Model C includes Model B and cardiovascular comorbidity (established coronary disease, heart failure and hypertension), smoking and GFR. With the Bonferroni correction, the results of patients without diabetes were not statistically significant (corrected P-level < 0.006 was not satisfied).

### Long-term mortality

There was a significant interaction between patients with and without diabetes as regards long-term mortality (*P* < 0.0001). In patients with diabetes, a significantly lower mortality risk was found in the BMI interval ≥30 kg/m^2^ in all three models (Table 4), also statistically significant after the Bonferroni correction (Bonferroni adjusted *P*-level < 0.001). In patients without diabetes and BMI < 18.5 kg/m^2^, the results showed a statistically borderline higher risk after the Bonferroni correction (Bonferroni-adjusted *P*-level = 0.006).

**Table 4:** The associated risk between non-normal BMI and mortality after the total follow-up time in the study (after 2.1 years, ± 1.5 years) in patients with or without diabetes seeking care at a hospital emergency department.

BMI categories	Patients with diabetes (HR, 95% CI)			Patients without diabetes (HR, 95% CI)		
	Model A	Model B	Model C	Model A	Model B	Model C
BMI < 18.5	0.67 (0.09–4.97)	0.95 (0.12–7.25)	1.32 (0.17–10.38)	1.67 (1.13–2.47)	1.64 (1.34–2.00)	1.76 (1.43–2.17)
BMI 18.5–24.9	Referent	Referent	Referent	Referent	Referent	Referent
BMI 25–29.9	0.66 (0.41–1.08)	0.83 (0.49–1.39)	0.82 (0.47–1.42)	0.86 (0.64–1.15)	0.88 (0.65–1.18)	0.82 (0.60–1.10)
BMI ≥ 30	0.41 (0.24–0.69)*	0.44 (0.26–0.75)*	0.40 (0.23–0.69)*	0.95 (0.68–1.31)	0.99 (0.72–1.37)	0.94 (0.67–1.31)

BMI: body mass index; CI: confidence interval; Hazard Ratio (HR):.

Analyses include imputed data for missing values for BMI.

Model A includes age and sex; Model B includes Model A and METTS-A triage; Model C includes Model B and cardiovascular comorbidity (established coronary disease, heart failure and hypertension), smoking and GFR. With the Bonferroni correction, the results in the diabetes group were statistically significant (corrected *P*-level < 0.006 was satisfied); however, in the non-diabetes group the values were borderline significant (corrected *P*-level = 0.006).

* *P* < 0.05 with the Bonferroni correction.

## Discussion

We found different patterns between individuals with and without diabetes as regards mortality in different BMI intervals. Regarding long-term mortality, patients with diabetes and BMI ≥ 30 kg/m^2^ showed a significantly lower risk than the reference, whilst patients without diabetes and underweight showed higher risk but only of borderline significance.

As regards diabetes patients with obesity, that is, BMI ≥ 30 kg/m^2^, the statistically significant lower risk are in line with the BMI–mortality paradox, that is, with lower mortality risks at higher BMI intervals, in contrast with results from large population studies showing an excess risk in the overweight and obesity interval (9, 21). In contrast, we could not find any statistically significant association between underweight and an increased mortality, but there were only two patients within this BMI interval.

One of the German studies of patients with type-2 diabetes revealed a U-shaped curve, with the lowest mortality rates for individuals with BMI at around 31 kg/m^2^ (22). Another study on patients with type 2 diabetes revealed a lower mortality rate among individuals with overweight and obesity class I (BMI 30–34.9 kg/m^2^) compared to individuals with normal BMI (23). A systematic review and meta-analysis confirmed these findings, with a higher mortality in underweight, that is, <18.5 kg/m^2^, and a lower mortality in overweight and mild obesity compared with normal BMI (24).

For patients without diabetes, we found no statistically significant results, but a borderline significance for underweight after the Bonferroni correction. Overweight and obesity were not associated with an increased mortality rate. Large studies have shown a U- or J-shaped curve, with the lowest BMI risk in individuals in the normal BMI interval (21). The research findings with a similar mortality in patients with overweight and obesity as in patients with normal weight thus in some respects could be seen as a marker of the obesity paradox. Underweight has been associated with an increased mortality in earlier, large population studies (9–11, 25). Furthermore, the association between underweight and increased mortality seems to be more pronounced in individuals with respiratory diseases (21). Actually, patients in this study actually had dyspnoea.

The obesity paradox has certainly been questioned. One of the reviews on the obesity paradox concluded on studies supporting this hypothesis: ‘these studies have numerous limitations due to their mainly retrospective nature and to numerous confounding factors, such as associated pathologies, antidiabetic treatments, smoking habit, lack of data about distribution of body fat or weight history’ (16), and another review on the same topic shows ‘conflicting evidence for the role of overweight and obesity in all-cause mortality may largely be a result of differences in study populations, epidemiological methods, and statistical analysis’ (15).

The reason for findings related to the obesity paradox has been discussed. Amongst patients with congestive heart failure, one of the studies found that increasing BMI was associated with lower mortality (26), especially for in-hospital mortality (27). Another study found the obesity paradox to be present, however, not in patients with diabetes (28), contradictory to the study findings. Yet, another study confirmed not only the association between higher BMI and lower mortality in patients with heart failure but also an association between being underweight and higher mortality (29). However, this study also concluded that the influence is complex, and that the effect of BMI was depending on the ejection fraction and the presence or absence of COPD (29). In contrast, for patients with COPD, obesity was associated only with mortality from respiratory causes and only in the BMI interval > 40 kg/m^2^ (30).

Excess mortality in low BMI could be explained by cancer mortality or smoking habits, but adjusting for this or by categorising into causes of mortality has shown that the BMI–mortality paradox still exists (12). Smoking has been shown to increase the mortality risk in the lower BMI interval (9). For patients with COPD, underweight is shown to be associated with increased mortality (30).

The patients in this study represent a special group, that is, patients seeking care at a hospital ED with symptoms of dyspnoea, and within this specific group it might be more dangerous being to lean. An earlier study in patients with acute MI found that excess weight at the time of MI was associated with a lower mortality, whilst in the long term it was associated with recurrent re-infarction and cardiac death (31). A review also confirmed the obesity paradox in MI, but also finding that this was true for long-term mortality (32), in contrast to other studies.

Thus, in general, there are contradictory findings related to the obesity paradox. An increased risk with underweight is regularly shown (9, 21); however, the relation between higher BMI intervals, that is, overweight and obesity, and mortality differs between studies, thus seemingly supporting the critical views in some reviews (15, 16). More studies on the possible effects of the obesity paradox in specific groups are warranted.

There are some limitations with this study. This is an observational study, and we had data only at baseline and not at follow-up. The sample was rather small with a small number of deaths, with a low statistical power. However, the sample represents a specific group in a specific setting, that is, patients with dyspnoea seeking care at the ED. We have no data on individual BMI trends. Furthermore, we imputed BMI values, which could have distorted the results, especially in the diabetes group, and this is why the results should be interpreted with some caution. Furthermore, the number of underweight patients was rather small, especially in the diabetes group with only two patients, and the association with mortality was not statistically significant after the Bonferroni correction, even if the mortality rate was highest within this group. The main aim was originally to study the association between BMI and short-term mortality; however, we also added the total registered mortality. Furthermore, the research study was not designed to analyse the work or effect of emergency care on patient outcomes, as there are too many unknown variables for analysis. We cannot generalise to other patient- age- and ethnic groups. Strengths of the study include the longitudinal study design and the use of real-world clinical data from a well-characterised cohort consisting of acute ill patients.

## Conclusions

We found for diabetes patients with overweight or obesity a lower overall mortality, in line with the obesity paradox, whilst those without diabetes no increased mortality was found for obesity, and the higher risk for underweight showed borderline significance after the Bonferroni correction. Taken together, our data suggest that obesity in this specific patient group seems to have a protective effect on patients with diabetes, and with no increased mortality risk for patients without diabetes compared with those with normal weight.
